# Case Report: Small bowel metastasis from esophageal squamous cell carcinoma: a case series and systematic review of clinical characteristics, tropism, and survival outcomes

**DOI:** 10.3389/fimmu.2026.1891441

**Published:** 2026-08-31

**Authors:** Mingyu Zhou, Yunjun Liu, Huifang Huang, Xia Chen, Qiwen Huang, Shaowei Huang, Zhichao Yan, Yuyin Chen, Junming Lin, Jiayi Huang, Guankao Xie, Yating Hou, Yisheng Huang

**Affiliations:** 1 The First School of Clinical Medicine, Guangdong Medical University, Zhanjiang, Guangdong, China; 2 Department of Oncology, Maoming People’s Hospital, Maoming, Guangdong, China; 3 The First School of Clinical Medicine, Southern Medical University, Guangzhou, Guangdong, China; 4 Department of Pathology, Maoming People’s Hospital, Maoming, Guangdong, China; 5 Jinan University, Guangzhou, Guangdong, China

**Keywords:** differential diagnosis, esophageal neoplasms, intestinal obstruction, multimodal treatment, small bowel metastasis

## Abstract

**Background:**

Small bowel metastasis from primary esophageal carcinoma is a rare occurrence, typically indicative of advanced disease and often presenting with non-specific abdominal symptoms. Failure to consider this possibility in the differential diagnosis may lead to delayed or incorrect management. This article reports the case of a 60-year-old male with small bowel metastasis originating from esophageal squamous cell carcinoma. To better characterize this clinical entity, we also conducted a systematic review of 23 documented cases, analyzing their clinicopathological features, diagnostic approaches, treatment modalities, and prognostic outcomes.

**Case presentation:**

A 60-year-old man presented with small bowel obstruction, ultimately diagnosed as metastatic esophageal squamous cell carcinoma. Surgical resection was followed by combined immunotherapy and chemotherapy, yielding a partial response.

**Systematic review:**

We reviewed 23 published cases, highlighting a strong male predominance (82.6%), jejunal predilection, and a median survival of only 6 months post-diagnosis. Small bowel metastasis from esophageal carcinoma, though uncommon, represents a challenging clinical scenario often mimicking more common abdominal pathologies. Histopathological confirmation is essential for accurate diagnosis. Early detection and a multidisciplinary treatment approach may correlate with favorable survival trends in these advanced-stage patients.

**Conclusions:**

Small bowel metastasis should be considered in esophageal cancer patients with acute abdominal symptoms. Histopathological confirmation is crucial, and a multimodal approach may be associated with extended survival trends in selected patients.

## Graphical Abstract

**Figure:**
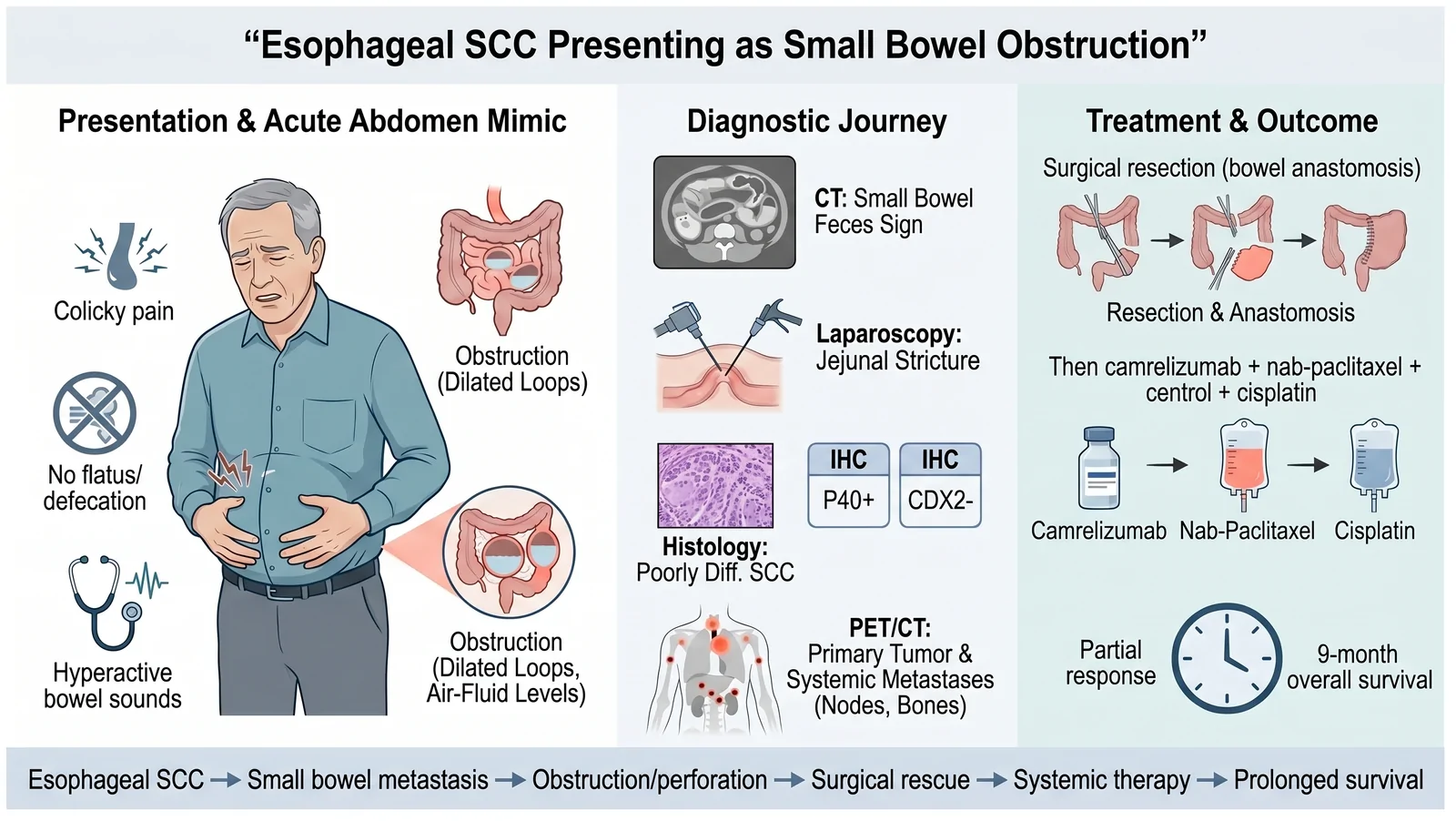
Multidisciplinary clinical pathway and management strategy for advanced esophageal squamous cell carcinoma presenting as an acute small bowel obstruction. The schematic outlines the patient*’*s diagnostic and therapeutic timeline divided into three sequential phases. (Left) Initial clinical presentation mimicking a standard acute abdomen emergency, characterized by colicky mid-abdominal pain, absolute constipation, hyperactive bowel sounds, and radiographic signs of mechanical small bowel obstruction. (Middle) The multimodal diagnostic journey, featuring an abdominal CT scan showing the small bowel feces sign, emergency laparoscopy revealing a tight jejunal stricture, histopathological and immunohistochemical analysis confirming metastatic squamous cell carcinoma (P40+, CDX2-), and whole-body PET/CT staging that localized the primary mid-esophageal lesion and widespread systemic metastases. (Right) The coordinated treatment strategy and oncological outcome, starting with emergency surgical resection and end-to-end anastomosis to resolve the mechanical crisis, followed by systemic chemoimmunotherapy consisting of camrelizumab, nab-paclitaxel, and cisplatin, which achieved a durable partial response and an overall survival of 9 months. SCC, squamous cell carcinoma; CT, computed tomography; IHC, immunohistochemistry; PET/CT, positron emission tomography/computed tomography; Diff., differentiated.

## Background

Esophageal carcinoma (EC) is a highly aggressive malignancy with a formidable global health burden, ranking eleventh in incidence and seventh in mortality worldwide (1). The predominant histologic subtype in Eastern Asia, including China, is squamous cell carcinoma (SCC), which is notorious for its propensity for metastasis (2). The unique anatomical features of the esophagus—specifically the lack of a true serosal layer (3) and a rich longitudinal lymphatic network—predispose it to aggressive local invasion and “skip” distant metastases (4). The common sites of distant dissemination are well-documented, primarily involving the liver, lungs, and bones, which are routinely screened during staging and follow-up (5).

However, the established metastatic map of esophageal cancer remains incomplete. The small intestine, despite being the longest segment of the gastrointestinal tract, is rarely considered a potential target for esophageal SCC metastasis (6, 7). This oversight creates a significant “diagnostic blind spot” in clinical practice. When patients present with acute abdominal emergencies such as intestinal obstruction, perforation, or bleeding, the clinical picture overwhelmingly points toward primary intestinal pathologies, benign causes, or other common abdominal malignancies. Consequently, the possibility of a metastatic lesion from an occult or known esophageal primary is frequently overlooked, leading to diagnostic delays and potentially inappropriate management strategies in the critical acute setting. The existing literature on this phenomenon is sparse, limited largely to isolated case reports (8–29), which precludes a comprehensive understanding of its clinical behavior and outcomes.

Therefore, this study aims to address this critical knowledge gap by presenting a highly instructive case of small bowel metastasis from esophageal SCC that initially manifested as intestinal obstruction. More importantly, we supplement this case with a systematic review of the available literature to aggregate existing evidence. Our objectives are to comprehensively delineate the clinicopathological characteristics, diagnostic challenges, treatment patterns, and prognostic profiles of this unusual metastatic pathway. By synthesizing evidence from our case and the literature, this report seeks to heighten clinical awareness and provide a practical diagnostic framework, ultimately aiding clinicians in the timely recognition and improved management of this elusive and aggressive disease complication.

## Case presentation

On July 21, 2022, a 60-year-old male was admitted to the Department of Gastroenterology at Gaozhou People’s Hospital, presenting with acute colicky mid-abdominal pain, abdominal distension, and a complete absence of flatus and defecation for half a day. Physical examination upon admission revealed stable vital signs, an acute illness appearance, mid-abdominal tenderness, and hyperactive bowel sounds, with an Eastern Cooperative Oncology Group (ECOG) performance status score of 2. The patient had no significant past medical history. Emergency upright abdominal radiography (**Supplementary Figure 1A**) suggested mechanical small bowel obstruction, characterized by multiple dilated small bowel loops and stepwise air-fluid levels in the mid-abdomen. Subsequent axial non-contrast abdominal CT (**Supplementary Figure 1B**) obtained on July 22, 2022, confirmed the obstruction, showing dilated small bowel loops (outer diameter > 2.5 cm) with the small bowel feces sign. While mesenteric fat planes were clear and vascular torsion was absent, initial imaging identified no definitive cause of the obstruction. Following the failure of conservative medical interventions, the patient was transferred to the Department of Gastrointestinal Surgery at Guangdong Provincial Second People’s Hospital on August 9, 2022, for emergency surgical evaluation.

During the preoperative diagnostic workup, serum tumor marker testing showed a significant elevation in cytokeratin 19 fragment (CYFRA 21-1, 27.02 ng/mL) and a mild elevation in carbohydrate antigen 15-3 (CA 15-3, 35.67 U/mL), while other markers including CEA, CA 19-9, AFP, and SCCA were within normal limits. The marked elevation of CYFRA 21-1, a specific marker for squamous cell carcinoma (SCC), raised a strong suspicion of malignancy of this histological type. Given the refractory nature of the obstruction, emergency laparoscopic surgery was performed, during which a localized, rigid, and stenotic segment was identified in the jejunum. Resection of the lesion-bearing small intestinal mass with end-to-end anastomosis was performed, and a biopsy of a suspicious abdominal wall nodule was conducted concurrently.

Pathological assessment of the resected specimens demonstrated multifocal carcinomatous infiltration. Gross examination of the small intestinal specimen (**Supplementary Figure 2A**) revealed a firm mural mass with transmural thickening (5 × 2.5 × 1 cm) and discrete pinhead-sized metastatic nodules on the serosal surface. Low-power hematoxylin-eosin (H&E) staining (**Supplementary Figure 2C**) showed a clear demarcation between normal mucosa and diffusely infiltrative tumor cells effacing the bowel wall architecture. High-power H&E staining (**Supplementary Figure 2B**) identified sheets of poorly differentiated pleomorphic tumor cells with frequent mitotic figures and focal squamous differentiation, such as intercellular bridges and abortive keratinization—diagnostic features of SCC.

To determine the tumor’s origin, an extensive immunohistochemistry (IHC) panel was performed. The neoplastic cells showed strong, diffuse cytoplasmic positivity for pan-cytokeratin (pan-CK, **Supplementary Figure 3A**) and diffuse, intense nuclear immunoreactivity for P40 (**Supplementary Figure 3B**) and P63 (**Supplementary Figure 4A**), confirming a squamous lineage. Conversely, the cells were negative for CDX-2 (**Supplementary Figure 3D**) and CD20 (**Supplementary Figure 4B**), effectively ruling out primary gastrointestinal adenocarcinoma and lymphoma. While CK7 showed focal moderate positivity (**Supplementary Figure 3C**), markers for other sites such as TTF-1, PAX-8, and PSA were negative. Additionally, p53 expression was null, and the Ki-67 proliferation index was approximately 40%. Next-generation sequencing (NGS) identified multiple genetic alterations, including amplifications and pathogenic mutations, within the MET, CCND1, VEGFA, and VEGFR2 pathways, while HER2 and EGFR profiles were wild-type. Immunotherapy-related testing revealed a PD-L1 combined positive score (CPS) of 0 (evaluated using the 22C3 pharmDx assay, Surexam company) and stable microsatellite status (MSS).

**Figure 1:**
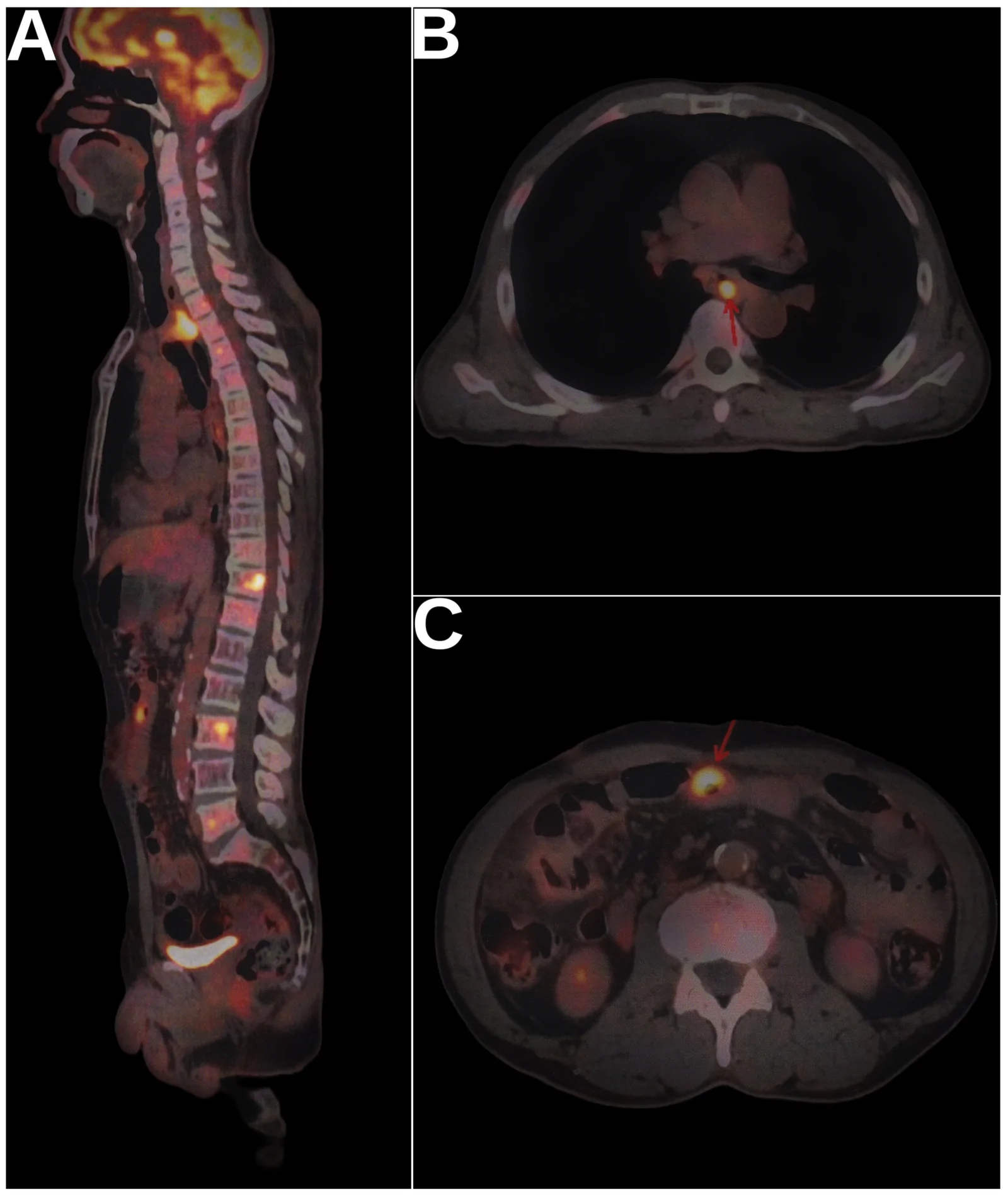
Whole-body PET/CT for primary tumor localization and staging **(A)** Axial fused ^18^F-FDG PET/CT at T6 level showing a mid-esophageal soft-tissue nodule (0.8×0.9×2.2 cm) with increased uptake (SUV_max_=6.0), consistent with primary esophageal squamous cell carcinoma. **(B)** Axial fused PET/CT at the jejunal anastomosis showing circumferential thickening (1.7×1.4 cm) with mild uptake (SUV_max_=5.3), likely post-surgical inflammation. **(C)** Maximum intensity projection (MIP) image revealing widespread systemic metastases: hypermetabolic supraclavicular, hilar, mediastinal, subdiaphragmatic and retroperitoneal lymph nodes (SUV_max_=5.9); diffuse osseous metastases (scapula, ribs, vertebrae, pelvis, femurs); and a suspicious hepatic lesion in segment S8 (SUV_max_=2.2). (All acquired August 25, 2022.).

On August 25, 2022, the patient underwent whole-body 18F-fluorodeoxyglucose (18F-FDG) PET/CT to locate the primary tumor. Axial fused images (**Figure 1A**) revealed a soft-tissue nodule in the mid-esophagus at the level of the 6th thoracic vertebra with increased FDG uptake (SUVmax = 6.0), consistent with primary esophageal SCC. Given the patient’s recent recovery from an acute intestinal obstruction, an invasive endoscopic ultrasonography (EUS) was not feasible; therefore, the primary lesion was clinically staged as cT1 based on the lack of deep adventitial invasion or bulky local disease on the cross-sectional imaging. Although the jejunal anastomosis showed reactive inflammation (**Figure 1B**), the maximum intensity projection (MIP) image (**Figure 1C**) confirmed extensive systemic metastases, including bilateral supraclavicular, hilar (SUVmax = 5.9), mediastinal, and retroperitoneal lymph nodes, as well as diffuse osseous metastases involving the ribs, vertebrae, and pelvis. While isolated hilar enlargement in elderly patients can be reactive, the significant hypermetabolic activity observed here, combined with concurrent widespread systemic dissemination, strongly indicated metastatic involvement rather than a benign inflammatory process. The final diagnosis was stage IV (cT1N2M1) poorly differentiated esophageal squamous cell carcinoma, classified according to the 8th edition of the American Joint Committee on Cancer (AJCC) staging system.

Systemic therapy was initiated following recovery from a recurrent obstruction that occurred on September 7, 2022, which was managed conservatively. The immunochemotherapy regimen consisted of the PD-1 inhibitor camrelizumab (200 mg, administered intravenously every 3 weeks), combined with nab-paclitaxel (150 mg) and cisplatin (37.5 mg). The chemotherapy agents were initially administered on a weekly schedule for the first 3 weeks. Subsequently, the treatment interval was adjusted, and the patient received an additional 4 cycles of the same drug combination administered on a standard 3-week (21-day) cycle. Remarkably, the systemic treatment was exceptionally well-tolerated. The patient did not experience any notable treatment-related adverse events, such as myelosuppression or gastrointestinal toxicity. Furthermore, no reactive cutaneous capillary endothelial proliferation (RCCEP), a characteristic adverse event associated with camrelizumab, was observed during the treatment course. Follow-up CT scans at three and six months demonstrated a partial response (PR) according to the Response Evaluation Criteria in Solid Tumors (RECIST) version 1.1 (30), with significant regression of the esophageal lesion and metastatic lymph nodes. Unfortunately, due to financial constraints and resistance to continued intravenous treatment, the patient was transitioned to palliative capecitabine monotherapy. Capecitabine was administered orally at a dose of 1000 mg/m² twice daily on days 1 to 14 of a 21-day treatment cycle. For this patient (body weight: 49 kg, height: 167 cm, body surface area: 1.51 m²), the calculated single dose was 1510 mg twice daily, and the practical single dose was adjusted to 1500 mg twice daily in accordance with the commercial tablet specifications. Unfortunately, due to severe financial constraints and the family’s decision to pursue exclusive home-based palliative care, no subsequent CT imaging was performed to formally evaluate the treatment response during this monotherapy phase. The patient ultimately succumbed to continuous disease progression, resulting in severe tumor-related cachexia and multiorgan failure, and died on May 6, 2023, with an overall survival of 9 months from the initial diagnosis.

**Figure 2:**
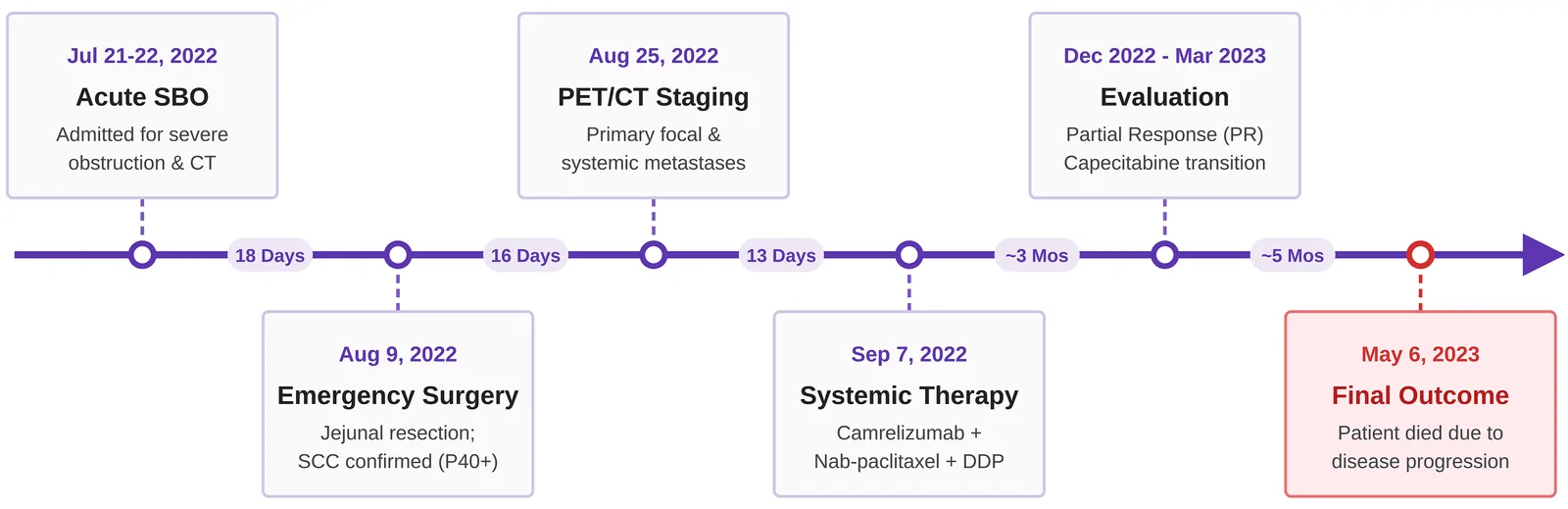
Comprehensive clinical timeline of the patient’s disease trajectory and management. The timeline chronologically illustrates the critical clinical milestones, beginning with the acute presentation of mechanical small bowel obstruction (SBO). It highlights the rapid sequence of diagnostic and therapeutic interventions, including emergency resection, pathological staging via PET/CT, and the initiation of systemic immunochemotherapy (camrelizumab combined with nab-paclitaxel and cisplatin). The clinical efficacy evaluations and the ultimate survival outcome are also documented. Exact dates are provided to demonstrate the real-world multidisciplinary management process. SBO, small bowel obstruction; SCC, squamous cell carcinoma; PET/CT, positron emission tomography/computed tomography; PR, partial response.

The comprehensive clinical course and management trajectory of the patient, from initial presentation to final outcome, are summarized in a timeline (**Figure 2**).

## Literature review

### Literature search strategy

A systematic literature search was conducted to identify all reported cases of small bowel metastasis from primary esophageal carcinoma. The databases of PubMed, Web of Science, and Google Scholar were comprehensively searched using the terms: (“esophageal cancer” OR “esophageal carcinoma” OR “esophageal neoplas*”) AND (“small intestine” OR “small bowel” OR “jejun*” OR “ile*”) AND (“metasta*” OR “secondary”). The references of retrieved articles were also manually screened for additional cases. No restrictions on publication date or language were applied. This search yielded 22 documented cases which, combined with the present case, provided a cohort of 23 for qualitative and quantitative synthesis (**Supplementary Table 1**), with the collective analysis of these cases offering a comprehensive visual summary of this rare metastatic pattern in **Supplementary Table 1**.

### Demographic and primary tumor characteristics

**Figure 3:**
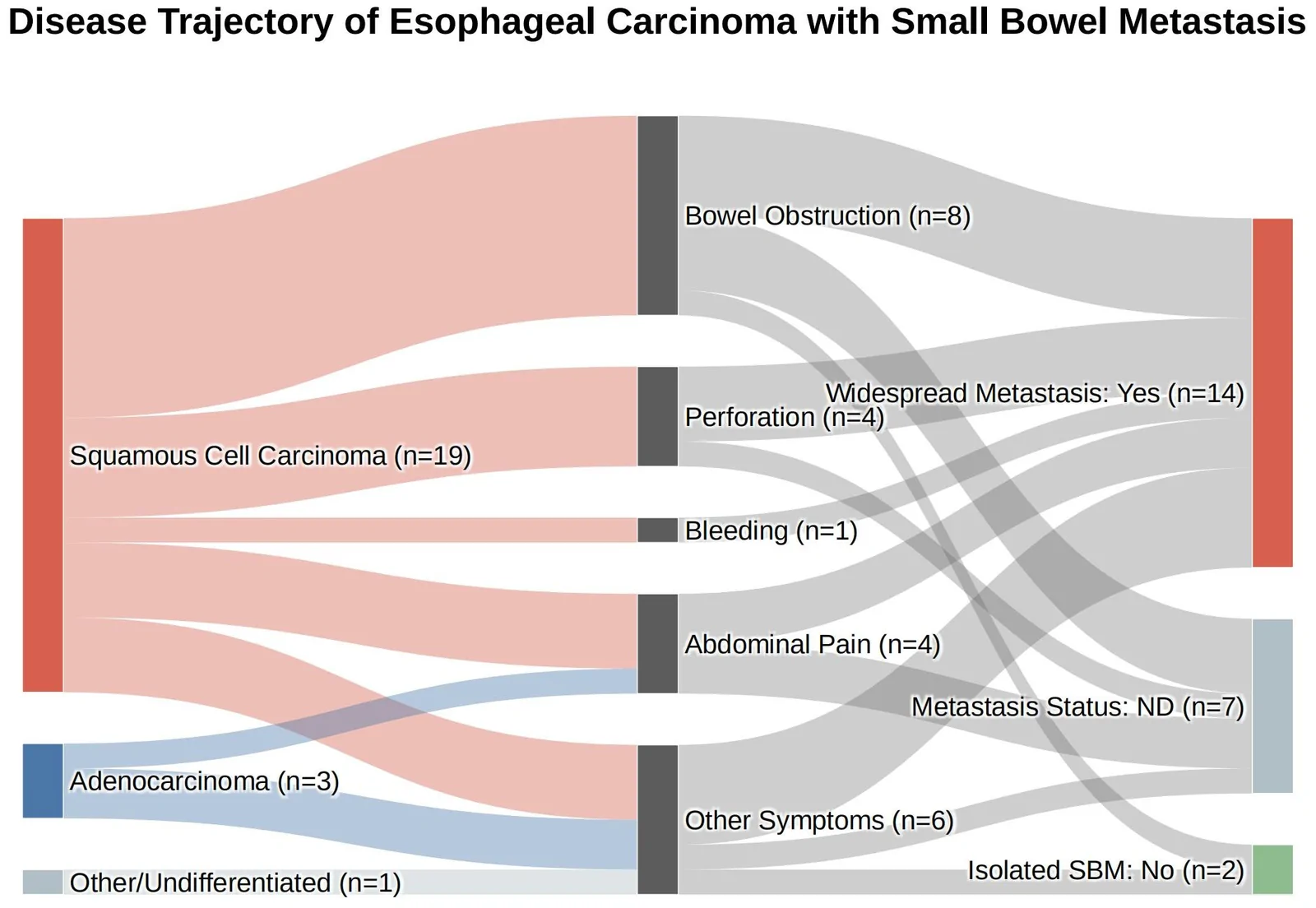
Sankey diagram depicting the disease trajectory and clinical characteristics of esophageal carcinoma with small bowel metastasis. Each horizontal line represents an individual patient’s survival trajectory (in months) based on available literature and the present case (n = 11), starting from the zero-point anchor. The colors indicate the treatment modalities: the red line highlights the present case (systemic therapy), dark blue lines represent literature cases receiving systemic therapy (e.g., chemotherapy, immunotherapy, or chemoradiotherapy), and light gray lines represent literature cases receiving surgery or conservative management only. Markers at the end of the lines indicate the final documented clinical status: crosses (X) represent confirmed death, right-pointing arrows (>) indicate that the patient was alive at the last follow-up, and vertical lines (|) indicate that the ultimate clinical outcome was unspecified in the original literature despite a documented follow-up duration. Cases completely lacking survival data were excluded from this plot.

As summarized in **Table 1**, the analysis included 23 patients with a pronounced male predominance (19 males, 82.6%). The median age at diagnosis of small bowel metastasis was 62 years (range: 41-86). The primary esophageal tumor was most frequently located in the middle (34.8%) and lower (34.8%) segments. Histopathologically, squamous cell carcinoma (SCC) constituted the overwhelming majority (19/23, 82.6%), while adenocarcinoma accounted for only 13.0% (3/23) of cases (8, 9, 16). The visual representation of this disease trajectory strongly emphasizes the unexpected propensity of SCC to metastasize to the abdominal cavity, challenging traditional assumptions regarding organ tropism (**Figure 3**).

**Table 1:** Clinical and demographic characteristics, management, and outcomes of patients with esophageal carcinoma and small bowel metastases.

Background data	Total cases (n = 15)
Background Data	Total Cases (n = 23)
Age, years, median (range)	62 (41–86)
Male, gender, n (%)	19 (82.6)
Primary esophageal cancer site, n (%)	
Middle segment	8 (34.8)
Lower segment	8 (34.8)
Gastroesophageal junction	3 (13.0)
Not specified	4 (17.4)
Primary esophageal cancer pathology, n (%)	
Squamous cell carcinoma	19 (82.6)
Adenocarcinoma	3 (13.0)
Other	1 (4.3)
Initial treatment with surgery, n (%)	13 (56.5)
Small bowel metastasis presentation, n (%)	
Bowel obstruction	8 (34.8)
Perforation	4 (17.4)
Bleeding	1 (4.3)
Abdominal pain	4 (17.4)
Other	6 (26.1)
Small bowel metastasis lesion count, n (%)	
Solitary	15 (65.2)
Multiple	6 (26.1)
Not reported	2 (8.7)
Treatment for small bowel metastasis, n (%)	
Surgical resection	17 (73.9)
Conservative management	3 (13.0)
Not reported	3 (13.0)
Metastases to other sites, n (%)	
Yes	14 (60.9)
No	2 (8.7)
Not reported	7 (30.4)
Survival after small bowel metastasis, months, median (range)	6 (1–9)^†^

Categorical variables are presented as counts and percentages [n (%)]. Continuous variables are presented as medians and ranges. †: Calculated based on the subset of patients with available survival data.

### Metastatic pattern and clinical presentation

The clinical presentation of small bowel metastasis was predominantly acute and severe. Intestinal obstruction was the most common manifestation, occurring in 34.8% of patients, followed by perforation (17.4%) and abdominal pain (17.4%). As depicted in the Sankey diagram (**Figure 2**), the clinical flow converges heavily on these surgical emergencies. Furthermore, the occurrence of small bowel metastasis often heralded a complete systemic collapse rather than an isolated event; 60.9% of patients presented with concurrent widespread metastases to other sites, most commonly the lymph nodes, liver, lungs, and bones.

### Diagnostic and therapeutic approaches

Diagnosis was predominantly established postoperatively, with surgical resection of the small bowel lesion serving as both a diagnostic and therapeutic intervention in 73.9% of cases. Computed tomography (CT) was the primary preoperative imaging modality, successfully identifying the acute abdominal complication but rarely specifying its metastatic nature beforehand. Immunohistochemistry (IHC) played a crucial role in confirming the esophageal origin; profiles such as CK7+/CK20-/CDX2- served as definitive molecular bridges linking the occult primary tumor to the intestinal metastasis, thereby ruling out primary gastrointestinal malignancies.

### Survival outcomes

**Figure 4:**
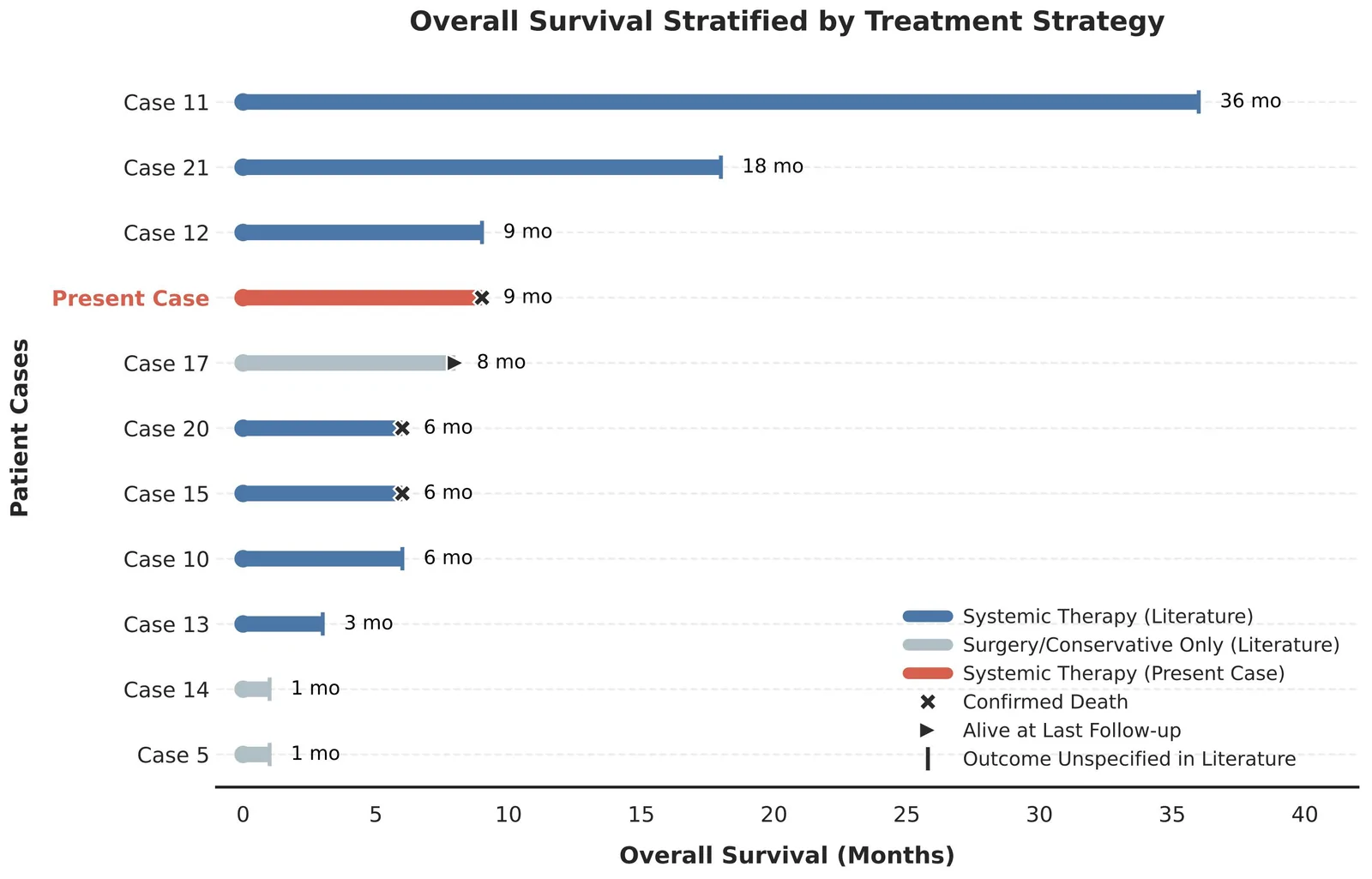
Swimmer plot illustrating the overall survival and treatment strategies of patients with small bowel metastasis from esophageal carcinoma.!!!Each horizontal bar represents an individual patient’s survival time (in months) based on available literature and the present case (n = 11). The colors indicate the treatment modalities: the red bar highlights the present case (systemic therapy), dark blue bars represent literature cases receiving systemic therapy (e.g., chemotherapy, immunotherapy, or chemoradiotherapy), and light gray bars represent literature cases receiving surgery or conservative management only. Markers at the end of the bars indicate the final survival status: crosses (X) represent confirmed death, whereas right-pointing arrows (>) indicate that the patient was alive or censored at the last follow-up. Cases with “Not Documented” survival times were excluded from this plot.

**Figure 5:**
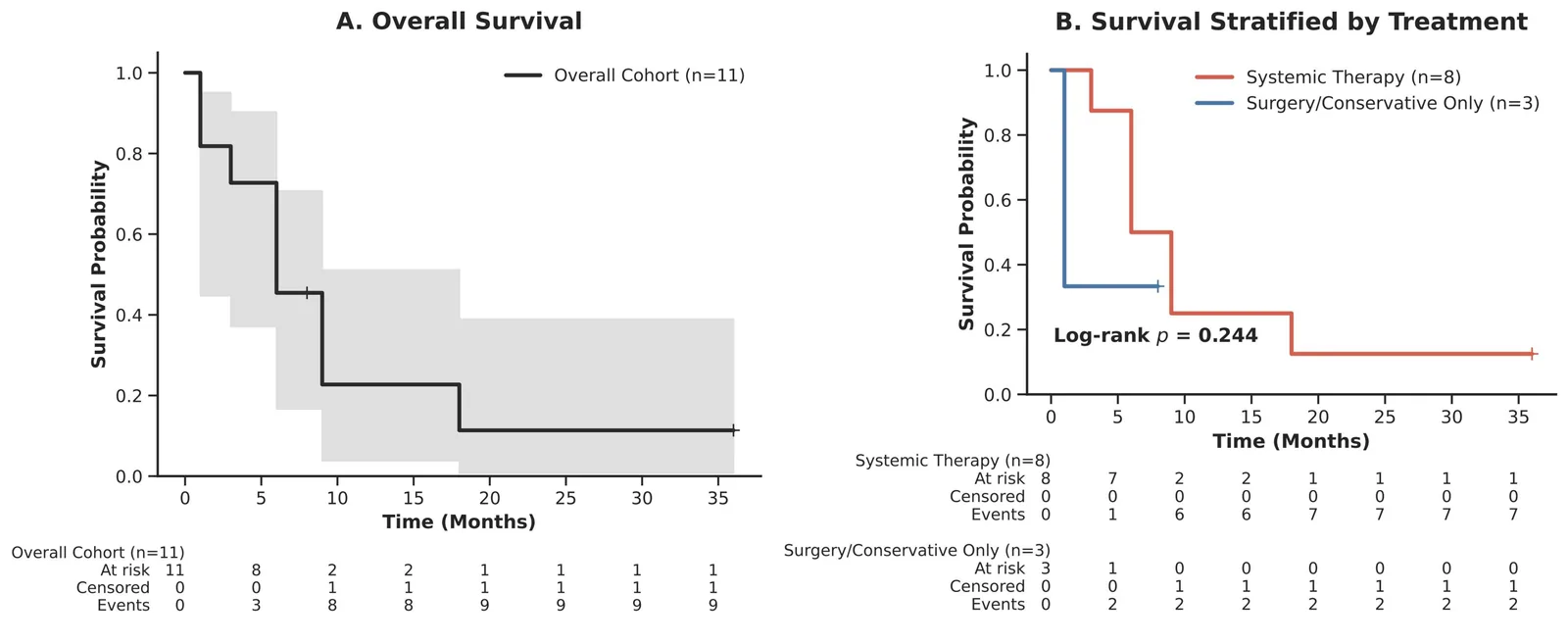
Kaplan-Meier estimates of overall survival for patients with small bowel metastasis from esophageal carcinoma. **(A)** Overall survival curve for the evaluable cohort (n = 11). The shaded area represents the 95% confidence interval. **(B)** Survival curves stratified by treatment strategy: patients receiving systemic therapy (n = 8, red line) versus those receiving surgery or conservative management only (n = 3, blue line). The systemic therapy group demonstrates a notable trend toward prolonged survival compared to the surgery/conservative group. Crosses (+) on the curves indicate censored observations (patients alive or lost to follow-up). The P value was calculated using the log-rank test. The tables below the x-axes display the exact number of patients at risk at the given time intervals.

The prognosis for patients with esophageal carcinoma and small bowel metastasis remains exceedingly poor. Among the pooled cohort, 12 cases lacked definitive survival information. Consequently, only 11 cases (including the present case) had definitive overall survival data available for quantitative analysis. As illustrated in the swimmer plot (**Figure 4**), the survival times exhibit significant heterogeneity, which is heavily influenced by the subsequent therapeutic strategy. Kaplan-Meier survival analysis revealed a sharp decline in overall survival within the first 6 months (**Figure 5A**). However, stratification by treatment modality unveiled a critical clinical trend (**Figure 5B**). Patients who received systemic therapy (such as chemotherapy, immunotherapy, or chemoradiotherapy) following the resolution of their acute abdomen demonstrated a notable trend toward prolonged survival compared to those receiving surgery or conservative management alone, whose survival rarely exceeded 2 months. This underscores that effectively managing the surgical emergency can provide a crucial window for systemic intervention, thereby extending the survival of this critically ill population.

## Discussion

In this study, we present a representative case of small bowel metastasis (SBM) from esophageal carcinoma (EC) and integrate it with the most comprehensive literature cohort to date (n=23) to systematically delineate the clinical profile of this rare entity. Our integrated analyses indicate that SBM represents a highly aggressive and prognostically poor late-stage event, rather than an incidental clinical outlier. Frequently presenting as acute surgical emergencies, SBM is associated with significant diagnostic challenges and complex therapeutic dilemmas. These findings provide valuable insights into the atypical biological behavior of advanced EC and underscore the necessity of optimizing multidisciplinary management strategies for this specific patient population.

Our aggregated data delineates a distinct clinical profile of EC with SBM: predominantly elderly males with esophageal squamous cell carcinoma (ESCC) who present with an acute abdominal crisis (e.g., obstruction or perforation). As our analysis indicates, these metastases are typically identified intraoperatively alongside widespread systemic dissemination, contributing to a poor baseline prognosis. These findings carry significant implications for acute care surgeons. When evaluating atypical, unexplained intestinal obstruction in middle-aged and older males, metastatic carcinoma should be considered in the differential diagnosis. The acute abdomen may represent the initial manifestation of an occult systemic malignancy. Intraoperative discovery of uncharacteristic small bowel masses necessitates prompt frozen section evaluation to avoid inappropriate surgical interventions and to facilitate accurate staging for subsequent oncological management.

While the precise anatomical route of SBM—whether via hematogenous micro-embolization, retrograde lymphatic spread, or transcoelomic seeding—warrants further investigation, the tumor biology of our cohort presents notable findings. Specifically, the strong predominance of Squamous Cell Carcinoma (SCC) (19/23, 82.6%) challenges the traditional “seed and soil” hypothesis of organ tropism (10, 11, 13–15, 17–29), which generally associates gastrointestinal adenocarcinomas with a higher affinity for intra-abdominal metastasis (6). As illustrated in the Sankey diagram (**Figure 3**), the SCC phenotype acts as the primary source, frequently progressing to mechanical crises (obstruction or perforation) and concurrent systemic metastasis. This visual trajectory suggests that a subset of ESCC may acquire specific molecular adaptations that facilitate colonization within the small intestinal microenvironment. It is important to note that these observations regarding distinct small bowel tropism and the propensity for aggressive transmural infiltration causing mechanical complications are drawn specifically from this SCC sub-cohort, and may not directly extrapolate to the adenocarcinoma cases. While recent high-impact studies have elucidated several key molecular drivers of general ESCC metastasis—such as the deregulation of SLIT2/Cdc42 pathways (31), the activation of Wnt signaling via NKD2 silencing (32), and the overexpression of receptor tyrosine kinases like EphA2, which has been shown to strongly correlate with lymph node metastasis and poor differentiation (33)—the precise mechanisms governing specific small bowel tropism remain undefined. Elucidating these unique molecular mechanisms could reveal novel therapeutic targets for intercepting this aggressive metastatic pattern.

The high incidence of mechanical obstruction (34.8%) (8–11, 13, 14, 18, 20, 22–24, 29) and perforation (17.4%) (14, 19, 21, 25, 26) in our cohort warrants a pathophysiological explanation. The transition from occult metastasis to an acute surgical crisis is driven by the specific growth patterns of ESCC within the intestinal wall. Histologically, metastatic squamous cell carcinoma often exhibits aggressive infiltrative growth through the muscularis propria, frequently accompanied by a pronounced desmoplastic reaction. This fibrotic response leads to transmural thickening, loss of bowel elasticity, and eventual luminal stenosis, clinically manifesting as mechanical obstruction. Furthermore, as the metastatic focus undergoes rapid proliferation that outstrips its local neoangiogenic vascular supply, the tumor core becomes highly susceptible to ischemic necrosis, ultimately culminating in transmural perforation. Understanding these histological behaviors bridges the gap between the tumor’s biological tropism and its catastrophic clinical presentation.

The diagnostic process for SBM frequently involves an occult primary tumor presenting initially through an acute abdominal crisis. In emergent settings, modern imaging modalities (such as CT) effectively identify the mechanical complication but often lack the specificity to determine its metastatic origin. Consequently, a comprehensive immunohistochemistry (IHC) panel is essential for accurate pathological diagnosis. Notably, p40 has been established in high-impact pathological consensus as a highly specific marker for squamous differentiation, significantly outperforming traditional p63 (34). As demonstrated in our case, the combination of intense p40 positivity and CDX2 negativity (a typical gastrointestinal epithelial marker) serves as a robust diagnostic panel (34). This specific immunoprofile provided definitive evidence linking an ambiguous intestinal stricture to its squamous esophageal origin, effectively excluding primary gastrointestinal adenocarcinomas or hematological malignancies.

The manifestation of SBM generally indicates stage IV disease, which historically carries a poor prognosis. However, our survival analyses offer a more nuanced perspective. The Swimmer Plot (**Figure 3**) reveals clinical divergence: patients receiving only surgery or conservative care experience rapid disease progression, whereas those who recover from the acute phase and subsequently receive systemic therapy exhibit prolonged survival. These data suggest a critical re-evaluation of the role of emergency surgery in SBM. Early clinical evidence from high-volume centers has highlighted that palliative surgical intervention is strongly justified for esophageal cancer patients presenting with gastrointestinal metastatic complications, especially in emergency scenarios involving obstruction or perforation (35). In this context, surgical resection acts as a pivotal bridging intervention designed to restore enteral function and physiological reserve, thereby enabling subsequent systemic therapy. While our case required open emergency surgery, the management of metastatic gastrointestinal tumors is increasingly influenced by super minimally invasive concepts (36), emphasizing that the goal of surgery in stage IV disease should be to maximize functional preservation and facilitate rapid recovery to minimize the interval to systemic treatment.

The treatment paradigm for advanced ESCC has recently undergone a revolutionary shift toward chemoimmunotherapy. Landmark trials such as ESCORT-1st (evaluating Camrelizumab) (37), CheckMate-648 (Nivolumab) (38), KEYNOTE-590 (Pembrolizumab) (39), and RATIONALE-306 (Tislelizumab) (40) have firmly established the overall survival benefit of adding PD-1 blockade to standard chemotherapy. Our case perfectly aligns with this modern oncological landscape. By successfully bridging the acute surgical crisis, the patient was able to receive a Camrelizumab-based immune-chemotherapy regimen, achieving a durable partial response and a 9-month overall survival. Furthermore, as organ preservation strategies become more prevalent for patients achieving a clinical complete response (cCR) after comprehensive therapy, clinicians must remain vigilant for distant relapses (7). The emergence of occult SBM highlights the necessity of expanding routine surveillance imaging beyond the thorax to include the entire abdomen, thereby eliminating diagnostic blind spots.

Our case aligns with the epidemiological and pathological hallmarks of this condition while highlighting the potential of modern oncological management. By integrating acute surgical intervention with precision medicine, it demonstrates that immune-chemotherapy regimens can yield meaningful clinical responses even in advanced SBM. It provides evidence that the historical median survival of 6 months may be extended when surgical and medical oncology strategies are effectively coordinated.

The manifestation of SBM generally indicates stage IV disease, which historically carries a poor prognosis. However, our survival analyses offer a more nuanced perspective. While overall survival remains limited (**Figure 5A**), the Swimmer Plot (**Figure 3**) reveals heterogeneity that appears associated with clinical intervention. The Kaplan-Meier stratification (**Figure 5B**) demonstrates a clinical divergence: patients receiving only surgery or conservative care experience rapid disease progression (typically <2 months), whereas those who recover from the acute phase and subsequently receive systemic therapy exhibit prolonged survival, occasionally extending beyond one year.

These data suggest a critical re-evaluation of the role of emergency surgery in SBM. Rather than serving solely as local palliation, surgical resection acts as a pivotal “bridging intervention.” Pathophysiologically, resolving the obstruction or perforation not only mitigates life-threatening sepsis but also restores enteral continuity and nutritional autonomy. This physiological rehabilitation, combined with the reduction of the macroscopic tumor burden (cytoreduction), is essential for enabling patients to tolerate the toxicity of subsequent systemic therapies. Our case illustrates the success of this multidisciplinary team (MDT) approach, where the surgical restoration of the gastrointestinal tract effectively paved the way for a combination of a PD-1 inhibitor (Camrelizumab) and chemotherapy, resulting in a durable partial response and a 9-month survival.

As a literature-based retrospective analysis, our study inherently faces several critical limitations. First, the pooled cohort of 23 patients was assembled from isolated case reports published across different decades, institutions, and geographic regions. This introduces substantial cohort heterogeneity, as the cases encompass varying histologic subtypes, span different staging eras with evolving diagnostic capabilities, and reflect significant shifts in treatment paradigms, most notably the recent introduction of immunotherapy. Second, as with most case-report-based literature, there is an inherent publication bias that favors the reporting of unusual clinical presentations and exceptionally favorable outcomes, which restricts the generalizability of our findings. Finally, the quantitative survival analysis is severely constrained by missing data, as 12 out of the 23 reviewed cases lacked documented survival outcomes. The extremely small evaluable sample size (n=11) dictated by the rarity of this condition and this high attrition rate limits the statistical power of our analyses. Moreover, our comparative survival observations are confounded by immortal-time bias, as patients must survive the initial acute surgical emergency to receive subsequent systemic therapy. Consequently, the pooled clinical features and survival trends should be interpreted strictly as exploratory, descriptive observations rather than definitive epidemiological or causal estimates, necessitating validation through future large-scale, multi-center cohort studies.

## Conclusions

In conclusion, small bowel metastasis from esophageal carcinoma is an aggressive late-stage event frequently presenting as an acute surgical emergency. Accurate diagnosis demands high clinical suspicion and definitive immunohistochemical profiling. Notably, the overwhelming predominance of squamous cell carcinoma challenges traditional views of organ tropism, revealing a distinct biological phenotype. Despite a dismal overall prognosis, our findings underscore the value of a proactive multidisciplinary approach. Emergency resection transcends terminal palliation to serve as a critical bridging intervention—resolving the immediate crisis to unlock a vital therapeutic window for modern systemic treatments, including immunotherapy, which may offer an exploratory trend toward prolonged survival.

## Data Availability

The original contributions presented in the study are included in the article/**Supplementary Material**. Further inquiries can be directed to the corresponding authors.
