# Supplementary material for: Case Report: Small bowel metastasis from esophageal squamous cell carcinoma: a case series and systematic review of clinical characteristics, tropism, and survival outcomes
Source: Front Immunol. 2026 Aug 31;17:1891441. doi: 10.3389/fimmu.2026.1891441 (PMC13572284; doi:10.3389/fimmu.2026.1891441)
Supplement: Supplementary file 1 [file Table1.docx]

Supplementary Table S1. Cases of esophageal carcinoma with small bowel metastasis in reviewed literatures.

| Case | Source | Gender | Age | Primary esophageal carcinoma | | | Metastatic lesion to small bowel | | | Metastases to other sites | Truncated/Overall survival |
| --- | --- | --- | --- | --- | --- | --- | --- | --- | --- | --- | --- |
|  |  |  |  | Site | Pathology | Therapy | Manifestation | Lesion count | Therapy |  |  |
| 1 | Chua et. al, 2021 [8] | Female | 41 | Gastroesophageal junction (GEJ), Siewert II | Moderately differentiated adenocarcinoma | ND | Obstructive jaundice, papillary stricture | 1 | ERCP with biliary stent | Multiple enlarged lymph nodes (GEJ region) | ND |
| 2 | Besaw et al., 2019 [9] | Female | 71 | Lower third of the oesophagus and gastro-oesophageal junction | Adenocarcinoma | FOLFOX + nivolumab; pembrolizumab | Severe abdominal pain, nausea, vomiting; dilated fluid-filled bowel | ND | Conservative management | Supraclavicular, celiac, hilar lymph nodes; lung | ND |
| 3 | Sreenarasimhaiah et al., 2005 [10] | Male | 62 | Esophagus (30-33 cm from incisors) | Squamous cell carcinoma | Palliative chemo/radiation | 2 cm mass in ampulla of Vater | 1 | ND | Regional lymph nodes | ND |
| 4 | Yang et al., 2025 [11] | Male | 67 | Lower esophagus (33-37 cm from incisors) | Squamous cell carcinoma | Abraxane + cisplatin + Sintilimab; radiotherapy | Hematochezia; abdominal/rectal masses with stenosis | 2 | Palliative care | Hepatogastric space lymph nodes | ND |
| 5 | Traill et al., 1997 [12] | Male | 46 | Distal oesophagus | Undifferentiated carcinoma | Palliative laser treatment | Small bowel ischemia; mesenteric venous thrombosis | 1 | Resection of ischemic bowel | Coeliac nodal mass, liver | 1 month |
| 6 | Wang et al., 1985 [13] | Male | 65 | Distal esophagus | Squamous cell carcinoma | Distal esophagectomy, esophagogastrostomy | Small bowel obstruction; jejunal mass | 1 | Resection of involved bowel | Recurrence at anastomosis site | ND |
| 7 | Neve et al., 2005 [14] | Male | 56 | Lower oesophagus (30-35 cm from incisors) | Squamous cell carcinoma | Transthoracic oesophagectomy; radiotherapy | Small bowel obstruction; ileal mass with perforation | 1 | Excision of ileal mass, ileostomy | Paraesophageal, perigastric lymph nodes | ND/ND |
| 8 | Lindenmann et al., 2005 [15] | Male | 54 | Middle third of esophagus (30-37 cm from incisors) | Squamous cell carcinoma | Transthoracic esophagectomy | Solitary submucosal jejunal nodule | 1 | Resection via jejunotomy | No other metastases detected | ND/ND |
| 9 | Huang et al., 2021 [16] | Male | 69 | Gastroesophageal junction (Siewert II) | Poorly differentiated adenocarcinoma | Neoadjuvant FLOT; Ivor Lewis esophagectomy | Three synchronous jejunal metastases | 3 | Resection during staging laparoscopy | Regional lymph node; brain metastasis | ND/ND |
| 10 | Yamana et al., 2013 [17] | Male | 69 | Lower thoracic esophagus (35 cm from incisor) | Squamous cell carcinoma (poorly differentiated) | Neoadjuvant chemo; thoracic esophagectomy | Solitary jejunal nodule | 1 | Partial jejunectomy | Paraesophageal lymph node; liver metastases | 6 months/ND |
| 11 | Yamada et al., 1996 [18] | Male | 56 | Middle esophagus | Squamous cell carcinoma | Distal esophagectomy; radiation; chemo | Small intestinal obstruction; jejunal mass | 1 | Resection of jejunal segment | Right chest wall metastasis | 3 years/ND |
| 12 | Chino et al., 2015 [19] | Male | 71 | Lower thoracic esophagus (35 cm from incisor) | Squamous cell carcinoma (moderately differentiated) | Neoadjuvant chemo; radical esophagectomy | Jejunal perforation with peritonitis | 1 | Partial jejunal resection, lavage | Multiple lymph node and bone metastases | 9 months/ND |
| 13 | Chandran et al., 2024 [20] | Male | 50s | Mid thoracic esophagus (30 cm from incisors) | Squamous cell carcinoma | Definitive chemoradiation | Ileal obstruction; omental nodules | 1 | Resection of ileal stricture | Mesenteric, omental nodules | 3 months/ND |
| 14 | Ono et al., 2018 [21] | Male | 86 | Middle thoracic esophagus (28 cm from incisors) | Squamous cell carcinoma (moderately differentiated) | None (patient declined) | Small intestinal perforation; submucosal nodule | 2 | Resection of bowel segment | Lung mass | 1 month/ND |
| 15 | Naoshima et al., 2021 [22] | Male | 60 | Lower thoracic esophagus | Squamous cell carcinoma (well-differentiated) | Neoadjuvant chemo; radical esophagectomy | Small bowel obstruction; two jejunal lesions | 2 | Resection of affected segment | Para-aortic, mesenteric nodes; peritoneal carcinomatosis | 6 months/ND |
| 16 | Lv Xuexia et al., 2010 [23] | Male | 65 | Not specified | Squamous cell carcinoma | Surgical treatment (2008) | Intestinal obstruction; jejunal mass | 1 | Resection of diseased intestine | No other sites mentioned | ND/ND |
| 17 | Zhang et al., 1993 [24] | Male | 48 | Esophagus (specific site not clear) | Esophageal squamous cell carcinoma | Not specified | Abdominal colic, pain, vomiting; ileal stenosis | 1 | Intestinal resection | Not mentioned | Good condition at 8 months |
| 18 | Liu et al., 2023 [25] | Male | 63 | Middle segment of esophagus (27-34 cm from incisors) | Moderately differentiated keratinizing SCC | Multiple chemo/immunotherapy regimens | Abdominal pain; ileal perforation with lumen narrowing | 1 | Partial ileum resection + anastomosis | Multiple sites: lung, lymph nodes, bones, etc. | ND/ND |
| 19 | Wang et al., 2017 [26] | Male | 73 | Middle segment of esophagus | Moderately differentiated SCC | Emergency surgery only | Acute abdomen; multiple small bowel lesions with perforation | 12 | Partial small intestine resection | ND | ND/ND |
| 20 | Liao et al., 2012 [27] | Female | 52 | Esophagus | Squamous cell carcinoma | Previous esophagectomy; chemotherapy | Periumbilical pain; polypoid jejunal tumor | 1 | Resection + chemotherapy | Died of metastasis | 6 months/ND |
| 21 | Li et al., 1999 [28] | Male | Not specified | Middle thoracic esophagus | Ulcerative squamous cell carcinoma | Multi-modality treatment | Abdominal pain, vomiting; ileal tumor with stenosis | 1 | Resection and anastomosis | Multiple lymph node metastases; local recurrence | 6 months/18 months |
| 22 | Liu et al., 2009 [29] | ND | ND | ND | Esophageal squamous cell carcinoma | Resection; chemotherapy | Ileal metastasis post-op; recurrent obstruction | 1 | Surgery + chemotherapy | ND | ND/ND |
| 23 | Present case | Male | 60 | Middle segment of esophagus (T6 level) | Poorly differentiated SCC (Stage IV) | Immunotherapy + chemotherapy | Intestinal obstruction; thickened bowel wall with serosal nodules | 4 | Laparoscopic resection + anastomosis | Widespread: lymph nodes, bones, liver, abdominal wall | ND/ND |
| Abbreviations: ERCP, Endoscopic Retrograde Cholangiopancreatography; FLOT, Fluorouracil, Leucovorin, Oxaliplatin, and Docetaxel; FOLFOX, Folinic acid, Fluorouracil, and Oxaliplatin; GEJ, Gastroesophageal Junction; MLSB, Metastasis to the Small Bowel; ND, Not Documented; PEC, Primary Esophageal Carcinoma; SCC, Squamous Cell Carcinoma. | | | | | | | | | | | |

Supplementary Figure S1. Clinical presentation at admission: mechanical small bowel obstruction. (A) Anteroposterior and lateral erect abdominal radiograph demonstrating mechanical small bowel obstruction. Multiple dilated small bowel loops with stepwise air‑fluid levels are seen in the mid‑abdomen; bowel wall is intact without free air. (B) Axial non‑contrast abdominal CT (soft‑tissue window) showing small bowel feces sign and dilated loops (>2.5 cm) with preserved mesenteric fat planes, excluding acute ischemia or closed‑loop obstruction. *(A) acquired July 21, 2022; (B) acquired July 22, 2022.*

Supplementary Figure S2. Gross and histopathologic morphology of the small intestinal tumor (A) Gross appearance of the resected specimen showing a firm transmural mass (5 × 2.5 × 1 cm) with focal mucosal erosion and discrete metastatic nodules in adjacent adipose tissue. (B) High‑power H&E (×200) revealing sheets of poorly differentiated pleomorphic tumor cells, high N/C ratio, frequent mitoses, and focal squamous differentiation (intercellular bridges, abortive keratinization). (C) Low‑power H&E (×100) demonstrating a sharp demarcation between normal mucosa and diffusely infiltrative tumor, with effacement of bowel wall architecture. *(A) acquired August 9, 2022; (B) and (C) from the same specimen.*

Supplementary Figure S3. Immunohistochemical phenotype confirming squamous cell lineage. (A) Diffuse cytoplasmic pan‑cytokeratin positivity confirms epithelial origin. (B) Intense nuclear P40 immunoreactivity, a specific marker of squamous differentiation. (C) Focal moderate CK7 positivity in scattered neoplastic cells, consistent with squamous cell carcinoma immunophenotype. (D) Complete nuclear negativity for CDX‑2, effectively ruling out a primary gastrointestinal origin. *(All images at ×200 magnification; internal positive/negative controls as described in original legends.)*

Supplementary Figure S4. Supplementary immunohistochemical markers for squamous differentiation and lymphoma exclusion. (A) Diffuse intense nuclear P63 positivity further supports squamous lineage differentiation. (B) Complete negativity for CD20 in neoplastic cells excludes primary or metastatic lymphoma. (Both images at ×200 magnification; from the same specimen as Figure S2 and Figure S3.)
